# Widding the Use of Insulin Sensitizers to Patients with Polycystic Ovarian Syndrome—A Late, but Wise Decision

**DOI:** 10.1055/s-0039-1685482

**Published:** 2019-03

**Authors:** Marcos Felipe Silva de Sá

**Affiliations:** 1 Department of Gynecology and Obstetrics, Faculty of Medicine, Universidade de São Paulo, Ribeirão Preto, SP, Brazil

Polycystic Ovarian Syndrome (PCOS) is the leading endocrine disorder in women of childbearing age with a prevalence of more than 15% among this group.^1^ These women are predisposed to obesity, have a predominance of the abdominal obesity phenotype associated with the hyperandrogenic state, insulin resistance (IR) and compensatory hyperinsulinemia.^2^ ^3^ It is estimated that 80% of women with PCOS have IR, and this scenario worsens in the presence of obesity.^4^ ^5^

A few authors suggest that there are abnormalities in the energy expenditure of women with PCOS, resulting from a reduction in the resting metabolic rate, especially in those with IR.^6^ In PCOS, has been considered a lower postprandial response of gastrointestinal hormones involved in neural control of food intake.^7^ ^8^ ^9^ ^10^ Food intake is controlled by complex interrelationships between homeostatic mechanisms that regulate caloric intake through a neuroendocrine system involving central and peripheral signals as well as mechanisms related to eating behavior. The central mechanisms are regulated by learning, memory and the reward system that acts in the mesolimbic circuit present in the central nervous system.^11^ ^12^ Gastrointestinal peripheral signs encompass several hormones that act on hunger (ghrelin) and satiation/satiety (cholecystokinin, YY peptide, oxintomodulin, glucagon like peptide - GLP-1, glucagon and amylin), and play an important role in regulating appetite and caloric intake.

Some studies have shown that the plasma levels of GLP-1 and PYY in the postprandial period are lower in women with PCOS,^13^ ^14^ and the exogenous administration of these hormones triggers the sensation of fullness and reduces caloric intake in animals and humans.^15^ Agonists of the GLP-1 have been used to promote weight loss in diabetic patients, and there is suggestion that these drugs could also benefit patients with PCOS.^16^ ^17^ ^18^ ^19^

Signs of body fat storage, such as insulin, leptin and adiponectin, also participate in the process of controlling food intake, as they indicate the state of energy reserve and alter appetite when necessary.^20^ Ghrelin is a gastrointestinal hormone known to stimulate food intake and its secretion is regulated by insulin levels and body reserves.^21^ ^22^ Women with PCOS have lower levels of fasting ghrelin and less postprandial suppression of ghrelin compared with obese women without PCOS.^7^ ^9^ A common finding in the literature is the negative correlation between insulin resistance and ghrelin plasma concentrations.^23^ ^24^ ^25^ When studying two groups of obese women (PCOS versus non-PCOS), we observed a lower ratio of preprandial ghrelin/insulin in the PCOS group. The postprandial ghrelin response was similar in both groups, and there was a significant negative correlation between ghrelin and insulin in both groups as well. However, when the patients were split into two groups, according to IR and regardless of PCOS, the negative correlation between ghrelin and insulin did not occur in the group with IR. These results suggest that the mechanism of ghrelin suppression by insulin may be impaired in women with IR, regardless of PCOS.^25^ Since IR is highly prevalent in obese PCOS patients, with rates above 90%,^4^ control of the satiation/satiety process is likely to function inadequately in these patients. In fact, in the patients we studied, an earlier increase in postprandial hunger was observed in obese women with PCOS than in obese women without PCOS. These observations have already been described by others,^7^ indicating the possibility that patients with PCOS would have impaired regulatory mechanisms of ingestion and satiation/satiety when compared with women without PCOS, which could explain the greater difficulty in weight loss of women with PCOS.

Weight loss has been the main option for treating PCOS. Low-glycemic diets and lifestyle changes associated or not to physical exercise lead to weight loss, reduce hyperandrogenism, increase ovulation and pregnancy rates, and are also beneficial in reducing hyperinsulinemia and its metabolic consequences.^26^ ^27^ ^28^ ^29^ ^30^ The main limitation of this type of therapy are the difficulties of maintaining weight loss for an extended period of time, and relapses are unfortunately frequent. Consequently, there is a growing use of bariatric surgery for the treatment of obesity in women with PCOS, which has significantly better results in terms of sustainable weight loss.^31^

The association of PCOS with IR, and, consequently, with hyperinsulinemia is known for decades, and in addition to its effect on the reproductive system, it is related to the high prevalence of obesity, glucose intolerance (GI), type 2 diabetes mellitus (DM2), dyslipidemia and vascular inflammatory processes. Consistent pictures with the metabolic syndrome, elevated triglycerides and LDL, and reduced HDL are frequent.^32^ ^33^

By considering these evidences, it is reasonable to propose that the main focus addressed in therapeutic strategies for weight loss in PCOS should also take into account the approach to IR. The use of insulin sensitizers reduces endogenous insulin requirements, with preservation of β cells, and it may protect insulin-resistant patients from developing DM2.^34^ ^35^

Despite the extensive literature on the deleterious role of IR in the evolution of PCOS and its association with changes in control mechanisms of caloric intake and satiation/satiety and the benefits of insulin sensitizers in disease therapy, important international societies have been reluctant to recommend its use associated with diet, lifestyle and physical exercise as the primary therapy of PCOS.

In the first consensus published as a group in 2004, even though the European Society of Human Reproduction (ESHERE) and the American Society of Reproductive Medicine (ASRM) recognized the high prevalence of IR in women with PCOS, they did not recommend IR screening in these patients by considering the restricted validity of clinical tests for its diagnosis. The group recognized some studies that showed the progression of GI to DM2 may be delayed by lifestyle changes and pharmacological intervention with insulin sensitizers, but did not recommend the use of these drugs routinely in the treatment of PCOS with IR.^1^

Four years later, the Thessaloniki ESHRE/ASRM-Sponsored PCOS Consensus Workshop Group proposed that “before any intervention is initiated, preconceptional counseling should be emphasized on the importance of lifestyle, especially weight reduction and exercise in overweight women, smoking, and alcohol consumption,” and recommended restricting the use of metformin (MTF) to women with PCOS and GI. Thus, the presence of IR should not be a reason for prescribing MTF.^36^

If IR is a step preceding GI, it did not seem reasonable to wait for the worsening of the metabolic profile in order to introduce insulin-sensitizing therapy. Considering the poor results obtained with lifestyle and diet changes on weight loss, we believe it is unreasonable to let the patient stay for a longer time under high levels of insulinemia and all its consequences.

However, the ESHERE and ASRM maintained the same position in a new version of the Amsterdam consensus published in 2011: “Diet and lifestyle are the first choice in improving fertility and prevention of diabetes (Level B). Metformin may be used for impaired glucose tolerance (IGT) and DM2 (Level A.).” Management of women at risk for DM2 should include diet and lifestyle improvement as first-line treatment. Treatment with MTF is indicated to those patients with IGT who do not respond adequately to calorie restriction and lifestyle changes.”^37^ The authors considered there is insufficient scientific evidence to recommend the use of insulin sensitizers beyond that situation, which indicates great caution in its use.

In a debate (pro x con) between two experts (Marshall and Dunaif),^38^ numerous justifications were offered for using MTF as an important adjuvant factor in the therapy of patients with PCOS, given the high prevalence of IR, obesity, GI, DM2, dyslipidemia, increased evidence of inflammatory process and metabolic syndrome.^38^ Marshal concluded that “the majority of evidence in adult women indicate that treatment of insulin resistance, either by lifestyle changes or metformin, leads to improvement in reproductive and metabolic abnormalities and probably reduces future development of diabetes and arterial disease.”^38^ Although Dunaif does not support the use of insulin sensitizers in all women with PCOS, she acknowledges this measure could be taken in view of the difficulties of maintaining diet and lifestyle in a younger population.^38^ In fact, several other societies have recommended the use of MTF in youngsters and adolescents with PCOS as a first-line therapy, combined or not with oral contraceptives and antiandrogens for the treatment of hyperandrogenemia and its symptoms, reestablishment of menses, aid with reduction of weight and IR, aiming at the prevention of long-term cardiovascular complications, even in lean adolescents.^39^ ^40^ ^41^ ^42^ ^43^ The fight against hyperandrogenism plays a key role in the process of containing the metabolic degeneration of these patients. The risk of GI and/or DM2 is greater in women who have oligo-anovulation and hyperandrogenism and this risk is even greater if they are obese.^44^

If the use of MTF has been practically a consensus for treating young, lean or obese, adolescents for some time, why not also apply it to adult women in the age group of 20 to 35 years, which corresponds to the prevailing age of patients with PCOS seeking care for the treatment of menstrual disorders and infertility? These are relatively young patients who may have their prognosis worsened and increased risk of metabolic syndrome if there is no intervention. Hence, it is reasonable to think that the benefits of insulins sensitizers could also reach this age group.

According to some authors, there are no data supporting the treatment of PCOS with MTF based on the measures of IR by arguing that the parameters for its calculation are not sensitive neither specific.^38^ However, other authors have contested this suggestion through the composition of various methods of calculations based on measurements of glucose and fasting insulin.^4^

Another argument against the use of MTF as first-line treatment for PCOS is the lack of data on the results of IR treatment per se in PCOS. Therefore, the metabolic assessment of women with PCOS should focus on detecting conditions that justify the intervention, such as GI, metabolic syndrome and elevated HDL levels.^38^ Metabolic syndrome and its indiviual components are common in patients with PCOS, particularly in those with elevated insulin levels (therefore, with IR) and increased weight. However, in patients with PCOS and normal body mass index (BMI), the diagnosis of metabolic syndrome is rare, although the literature shows that 19% of patients without metabolic syndrome may have GI,^32^ and, certainly, a much higher percentage may have IR, since it can be found in up to 70% of non-obese patients with PCOS.^4^ For example, is there any doubt about IR in patients with Acanthosis Nigricans, even if they are lean?

In fact, randomized clinical trials in women with PCOS are needed to check the efficacy of insulin sensitizers for the improvement of metabolic endpoints. The criticism of the literature is that much information was obtained in studies investigating non-metabolic endpoints in PCOS or that non-PCOS populations were studied.^36^ ^37^ ^38^ Randomized clinical trials are an essential tool in the construction of scientific evidence for clinical practice, although not always the most useful for some evaluations. Generally, their performance is complex, costly, with numerous operational difficulties, and many of them do not have adequate sample size for evaluating therapeutic practices. Moreover, although the findings of controlled therapeutic trials are statistically significant, there is no guarantee they will serve the totality of individuals. Considering the vast medical scenario, there are few situations in which decision-making is supported by evidence-based medicine. Most health practices are based on experimental cohort or case-control studies, or even non controlled observation of a set of cases.

In the specific case of PCOS, it is easy to imagine how difficult it would be to carry out a randomized clinical study to identify patients at risk and conduct a longitudinal study of cohorts of women with PCOS beyond 60 years of age to determine with greater precision the time and efficacy of the interventions studied for answering if insulin sensitizers used in patients with IR definitely prevent GI or DM2. Many patients would be followed up for decades and after entering their postmenopausal period.

The lack of these studies may mean we do not have definitive answers to elucidate endpoints. However, the lack of these answers should not be enough reason to stop prescribing those drugs for these patients. Numerous evidences based on experimental cohort or epidemiological studies also have their value. By exclusively valuing evidence-based medicine obtained in randomized clinical trials or systematic reviews with meta-analysis, we certainly fail to cover numerous clinical situations, as in the case of PCOS. In these situations, it is absolutely pertinent to use evidence based on medicine rather than evidence-based medicine for decision-making in the face of individualized clinical situations. Thus, studies with less rigorous designs may have their place in everyday practice for the care of a particular individual.^45^

Although the FDA has approved MTF for DM2 treatment, it has been used off label for more than 40 years to reduce the signs and symptoms of PCOS and normalize the various parameters that assess risks for DM2, obese and non-obese, children, adolescents and adults.^35^ ^41^ ^46^ ^47^ ^48^ ^49^ For these reasons, we have been asking ourselves: why not routinely prescribe it for hyperandrogenic and obese patients with PCOS, regardless of whether they have GI or DM2? Should we expect the worsening of the insulin resistance picture for its prescription?^50^

Finally, in the last ASRM/ESHERE consensus, it was recognized the validity of a more comprehensive use of MTF in the treatment of PCOS, based on evidence of its clear benefits in the specific PCOS group or subgroups by improving weight, BMI, hip-waist ratio, testosterone and glucose, especially in obese patients, since metabolic benefits are more accentuated in patients with increased BMI. Thus, in addition to lifestyle changes, the use of MTF was recommended in adult women with PCOS for the treatment of overweight and hormonal and metabolic changes. The benefit of MTF use in adolescents diagnosed with PCOS was also considered.^51^ Although not specified, note that a more comprehensive introduction of insulin sensitizers may also contribute to a better observation and comprehension of the effects of IR on the control mechanisms of ingestion/satiation/satiety that are possibly committed in PCOS.

Faced with so much evidence, restricting the use of insulin sensitizers for patients with PCOS no longer made sense. This new position of the most important societies of the specialty expands the range to patients with PCOS that can benefit from these drugs. The restriction on its use until recently may have deprived thousands of patients of the benefit of reducing IR and its consequences. It is known that IR is related to endothelial dysfunction and that it progresses with time. Endothelial dysfunction caused by IR has been related to reduced nitric oxide bioavailability and changes in endothelial regeneration. Research on the use of insulin sensitizers for the treatment of polycystic ovaries in rats has shown that endothelial dysfunction is the direct result of hyperandrogenism induced by IR, and treatment with MTF improves insulin sensitivity and blood pressure and reestablishes normal endothelial function, even with the weight gain of animals.^52^ Some studies have also shown that pioglitazone, an insulin sensitizer, has effects on glucose homeostasis, and exerts pleiotropic effects, thereby improving endothelial dysfunction.^53^

Professionals from all over the world follow guidelines of the major American and European specialty societies, which impact direct on the therapeutics adopted globally. Therefore, the wider use of insulin sensitizers brings great prospects of more concrete clinical results regarding this therapy for both young and adult patients with PCOS, especially obese patients, as a first-line treatment. We believe that gynecologists are able to prescribe this drug because international experience has shown this is a low-risk product that has been increasingly used in patients with PCOS, youngsters, adolescents and adults, obese or non-obese, including pregnant patients with PCOS and gestational diabetes, and in hypertension during pregnancy.^54^
